# Drinking carrot juice increases total antioxidant status and decreases lipid peroxidation in adults

**DOI:** 10.1186/1475-2891-10-96

**Published:** 2011-09-24

**Authors:** Andrew S Potter, Shahrzad Foroudi, Alexis Stamatikos, Bhimanagouda S Patil, Farzad Deyhim

**Affiliations:** 1 Department of Human Sciences, Texas A&M University-Kingsville, Kingsville, TX 78363, USA; 2 Department of Horticultural Sciences, Vegetable and Fruit Improvement Center, Texas A&M University, College Station, TX 77843, USA; 3 Citrus Center, Texas A&M University-Kingsville, Weslaco, TX 78596, USA

**Keywords:** Antioxidant status, cardiovascular disease, lipid peroxidation

## Abstract

**Background:**

High prevalence of obesity and cardiovascular disease is attributable to sedentary lifestyle and eating diets high in fat and refined carbohydrate while eating diets low in fruit and vegetables. Epidemiological studies have confirmed a strong association between eating diets rich in fruits and vegetables and cardiovascular health. The aim of this pilot study was to determine whether drinking fresh carrot juice influences antioxidant status and cardiovascular risk markers in subjects not modifying their eating habits.

**Methods:**

An experiment was conducted to evaluate the effects of consuming 16 fl oz of daily freshly squeezed carrot juice for three months on cardiovascular risk markers, C-reactive protein, insulin, leptin, interleukin-1α, body fat percentage, body mass index (BMI), blood pressure, antioxidant status, and malondialdehyde production. Fasting blood samples were collected pre-test and 90 days afterward to conclude the study.

**Results:**

Drinking carrot juice did not affect (P > 0.1) the plasma cholesterol, triglycerides, Apo A, Apo B, LDL, HDL, body fat percentage, insulin, leptin, interleukin-1α, or C-reactive protein. Drinking carrot juice decreased (P = 0.06) systolic pressure, but did not influence diastolic pressure. Drinking carrot juice significantly (P < 0.05) increased the plasma total antioxidant capacity and decreased (P < 0.05) the plasma malondialdehyde production.

**Conclusion:**

Drinking carrot juice may protect the cardiovascular system by increasing total antioxidant status and by decreasing lipid peroxidation independent of any of the cardiovascular risk markers measured in the study.

## Background

Despite a gradual decline in mortality from cardiovascular disease (CVD), it is still the leading cause of both morbidity and mortality in the United States [1]. Approximately 864,000 Americans die each year from CVD and this figure makes up 35% of the total deaths in the United States [2]. In recent years, there have been disturbing increases in the prevalence of CVD risk factors like diabetes, obesity, and the metabolic syndrome which collectively may negate the downward trends in CVD mortality [1,3,4].

Obesity elevates the risk of developing metabolic syndrome, diabetes, and CVD [5-7]. Obesity is thought to initiate a cascade of events leading to systemic inflammation and increases in circulating C-reactive protein, insulin resistance, and dyslipidemia [7]. In 1998, the American Heart Association considered obesity to be one of the major risk factors for coronary heart disease [8]. Other risk factors for CVD include a higher body mass index (BMI), the marker commonly used to establish obesity, which has been shown to be independently associated with hypertension, elevated total cholesterol and low-density lipoprotein (LDL) cholesterol levels, and lower high density lipoprotein (HDL) cholesterol [9-12]. The optimal BMI for adults 18 to 85 years of age is from 23 to 25 for most races [13]. Evidence has demonstrated that people with elevated BMI are at higher risk of developing CVD compared with those of normal BMI [14,15]. Additionally, the presence of metabolic syndrome is predicted to shorten life leading to death at younger ages [16].

Diets high in fat and cholesterol are the major factors contributing to CVD. Dietary modification and lifestyle changes are suggested to be effective strategies to prevent CVD [17]. The National Heart, Lung, and Blood Institute (NHLBI) recommend the Therapeutic Lifestyle Change (TLC) diet to improve heart health in individuals at risk for CVD. The TLC diet consists of reducing intake of saturated and total fat from animal products and increasing the intake of fibrous vegetables, fruits, whole grains, and legumes [18]. Increased fruit and vegetable consumption has been found to play a key role in preventing heart disease. In a follow up of the Nurse's Health Study, an additional serving per day of fruits and vegetables was associated with a 4% reduction in the risk of coronary heart disease [19]. Fruits and vegetables contain many nutrients which may be associated with reduced risk for heart disease, including fiber, vitamins, minerals, and phytochemicals. Phytochemicals found in fruits and vegetables have been shown to reduce inflammation, oxidative stress, and other markers of CVD [20].

Among common fruits and vegetables, carrots are high in fibers, carotenoids, vitamins C and E, and phenolics such as *p*-coumaric, chlorogenic, and caffeic acids [21]. Consuming foods containing phenolic compounds has decreased the risk of vascular diseases in previous studies [22,23]. Phenolic compounds are dietary antioxidants found in plants that are shown to inhibit LDL oxidation, inhibit platelet aggregation and adhesion, decrease total and LDL cholesterol, and induce endothelium-dependent vaso-relaxation [24-26]. Oral intake of carrot juice also displays other beneficial physiological effects including reduced oxidative DNA damage [27], increased levels of plasma antioxidants [28], and reduced inflammation [29]. In the Lipid Research Clinics Coronary Primary Prevention Trial (LRC-CPPT), men were tracked over 13 years and results revealed that those with the highest plasma carotenoid levels had lower risk of coronary heart disease [30]. In a 12-year follow-up of the Prospective Basel Study, Eicholzer and colleagues found that the risk of ischemic heart disease is increased by 1.53 Relative Risk in those with the lowest plasma carotene concentrations [31]. Inflammation has been shown to be a strong predictor of CVD and serum β-carotene inversely correlates with C-reactive protein and interleukin-6 [29,32].

Previous studies have shown beneficial effects of eating high fiber diets on lowering cardiovascular risk factors [33-35]. One other study has shown potential benefit of fruit and vegetable juice concentrate on cardiovascular prevention [36]. The goal of present study was to evaluate the potential role of drinking 16 ounces fresh squeezed carrot juice (equivalent to one pound of fresh carrot) daily on lowering cardiovascular risk markers in adults with elevated cholesterol and triglycerides.

## Methods

### Subjects

In this study, eight males and nine females from Texas A & M University-Kingsville (TAMUK) faculty and staff (n = 17) with elevated plasma cholesterol and triglyceride levels were the study participants. Each subject was asked to drink 16 fl oz fresh carrot juices daily for the three month duration of the study. The research study was approved by TAMUK IRB-board prior to initiation of the study. Participants agreed to drink the carrot juice daily and signed a consent form to agree to rules and regulations of the study. Carrots were juiced and delivered to research participants daily. Weight and height were measured both pre-test and post-test to calculate BMI status. Fasting blood samples were collected by a licensed nurse at the start of the study and after 90 days when the study was concluded. The blood samples were collected in tubes and centrifuged at 1,500x g for 15 min. The blood chemistry and lipid panels were analyzed using Modular Analytics D 2400 system and Cobas Integra 800 of Roche Diagnostics Corp. Indianapolis, IN.

### Outcome Measures

#### Blood Pressure Collection

Three random blood pressures from the right upper arm were taken using the OMRON Model #HEM 711. Blood pressure was measured at the beginning and end of the study. The blood pressure was taken from each subject and repeated if: 1) an error occurred in the reading, 2) the subjects seemed anxious or nervous, or 3) the blood pressure measurement was above the normal range (120 mm Hg/80 mm Hg).

#### Bioelectrical Impedance Analysis

The Bioelectrical Impedance Analysis (BIA) technique was used to estimate percentage of body fat using Quantum II (RJL Systems, 2006, Clinton Twp., MI). The BIA test was performed while each subject was lying supine with their arms and legs spread open. After the electrode site was cleaned with isopropyl alcohol, electrode patches with self-adhesive conducting gel were attached to the dorsal surface of the right foot and right hand. The electrodes introduced an alternating current (50 kHz) at the base of the toes and fingers and the Quantum II measures the voltage changes (RLJ Systems, 2006, Clinton Twp., MI). The BIA was measured at the beginning and end of the study.

#### Inflammatory Markers and Hormones

The plasma interleukin-1α was determined as a pro-inflammatory marker (R&D System, Minneapolis, MN). The plasma C-reactive protein was analyzed using a rat C-reactive protein ELISA kit as an index for inflammation (Life Diagnostics, Westchester, PA, USA). Insulin was analyzed to determine an insulin resistant state (Linco Research, Inc. St. Charles, MI). Leptin was analyzed as an adiposity hormone (Linco Research, Inc. St. Charles, MI).

#### Total Antioxidant Status and Malondialdehyde Production

The plasma was collected and an aliquot was refrigerated for total antioxidant status using a commercially available kit (Calbiochem, San Diego, CA, USA) as a quantitative measure of circulating antioxidant status. The plasma malondialdehyde was evaluated as an indicator of lipid peroxidation using a kit from Northwest life science (Vancouver, WA, USA).

### Analysis

The experimental design was a pre-test/post-test and a comparison student t-test was performed to determine the effects of drinking 16 fl oz fresh carrot juice daily as an independent variable on variables of interest at baseline and 90 days after the completion of the study as outlined by Steel and Torrie [37].

## Results

Drinking 16 fl oz of carrot juice daily for three months did not (P > 0.1) alter subject's weight, body fat percentage, or BMI (Table 1). While drinking carrot juice numerically lowered systolic pressure (P = 0.06) while diastolic pressure was unchanged (Table 1). The fasting plasma chemistry including glucose, triglycerides, cholesterol, HDL, LDL, VLDL, the hormones insulin and leptin, and the inflammatory markers interleukin-1α and C-reactive protein were unaffected from consuming carrot juice (Table 1). Plasma Apo A and Apo B were not (P > 0.1) significantly affected by drinking carrot juice (Table 1). However, the plasma antioxidant status was significantly (P < 0.05) increased while the plasma malondialdehyde production was significantly (P < 0.05) decreased after drinking carrot juice (Table 1).

**Table 1:** Effects of drinking carrot juice on anthropometrics, blood Pressure, blood chemistry, lipid panel, hormones, inflammatory markers, and antioxidant status

Variables	Pre-Test	Post-Test
**Anthropometric**		
Body weight (Kg)	83 ± 16	83 ± 16
Height (cm)	165 ± 2	165 ± 2
BMI	30 ± 2	30 ± 2
Fat %	28 ± 2	33 ± 2
**Blood Pressure**		
Systolic (mm Hg)	126.5 ± 2	120.5 ± 2
Diastolic (mm Hg)	77 ± 2	75 ± 2
**Blood Chemistry**		
Na (mmol/L)	140 ± 0.4	139 ± 0.4
K (mmol/L)	4.16 ± 0.07	4.32 ± 0.07
Ca (mg/dl)	9.4 ± 0.07	9.5 ± 0.07
Albumin (g/dL)	4.26 ± 0.06	4.30 ± 0.06
Glucose (mg/dL)	94 ± 1.9	93 ± 1.9
**Lipid panel**		
TAG (mg/dL)	115 ± 14	123 ± 14
Cholesterol (mg/dL)	212 ± 8	218 ± 8
HDL (mg/dL)	55 ± 5	54 ± 5
VLDL	30 ± 4	32 ± 4
LDL (mg/dL)	128 ± 7	133 ± 7
Apo A (mg/dL)	162 ± 9	153 ± 9
Apo B (mg/dL)	102 ± 5	103 ± 5
**Hormones**		
Insulin (μU/mL)	10 ± 1.1	9 ± 1.1
Leptin (ng/mL)	22 ± 2.8	21 ± 2.8
**Inflammatory markers**		
Interleukin-1α (pg/mL)	1.48 ± 0.13	1.82 ± 0.15
C-reactive protein (mg/L)	3.74 ± 0.43	2.75 ± 0.45
**Antioxidant status**		
Total antioxidant capacity (mM)	0.81 ± 0.04^b^	1.03 ± 0.04^a^
Malondialdehyde (μM)	42 ± 6^a^	18 ± 6^b^

^a,b^Means with unlike superscript are significantly (P ≤ 0.05) different from each other.

Comparing the males to the females, men weighed more and were taller than the female subjects (Table 2). The plasma chemistry, lipid panel, blood pressure, and plasma antioxidant capacity were not (P > 0.1) different between men and women participants (Tables 2 and 3). However, the plasma interleukin-1α, Apo B, and malondialdehyde were significantly (P < 0.05) higher in men (Table 3), while the percentage of body fat, insulin, and leptin were higher (P < 0.05) in women (Tables 2 and 3). It is interesting to note that at baseline, the men had significantly higher (P < 0.05) plasma malondialdhyde concentration than the women, but after drinking carrot juice for 90 days, the plasma malondialdhyde concentration was significantly reduced (P < 0.05) and there was no difference in the plasma malondialdehyde concentration between men and women participants at the end of the study.

**Table 2:** Gender differences on anthropometrics, blood Pressure, blood chemistry, and insulin

Variables	Female	Male
Body weight (Kg)		
Pre	75 ± 8^b^	90 ± 8^a^
Post	76 ± 5^b^	90 ± 8^a^
Height (cm)		
Pre	155 ± 1^b^	173 ± 1^a^
Post	157 ± 1^b^	173 ± 1^a^
BMI		
Pre	31 ± 2	30 ± 2
Post	30 ± 2	30 ± 2
Fat (%)		
Pre	41 ± 2^a^	18 ± 2^b^
Post	43 ± 3^a^	20 ± 3^b^
Systolic pressure (mm Hg)		
Pre	125 ± 3	127 ± 3
Post	118 ± 2	122 ± 2
Diastolic pressure (mm Hg)		
Pre	78 ± 2	75 ± 2
Post	75 ± 2	74 ± 2
Na (mmol/L)		
Pre	139 ± 0.6	140 ± 0.6
Post	128 ± 0.6	139 ± 0.6
K (mmol/l)		
Pre	4.14 ± 0.09	4.30 ± 0.11
Post	4.15 ± 0.09	4.40 ± 0.11
Ca (mg/dL)		
Pre	9.46 ±0.11	9.59 ± 0.11
Post	9.44 ± 0.07	9.32 ± 0.07
Albumin (g/dL)		
Pre	4.2 ± 0.08	4.3 ± 0.09
Post	4.2 ± 0.08	4.6 ± 0.09
Glucose (mg/dL)		
Pre	92 ± 3	96 ± 3
Post	93 ± 3	93 ± 3
Insulin (μU/mL)		
Pre	11.80 ± 1.25^a^	9.00 ± 1.30^b^
Post	11.72 ± 1.25^a^	7.80 ± 1.35^b^

^a,b^Means with unlike superscript are significantly (P ≤ 0.05) different from each other.

**Table 3:** Gender differences on lipid panel, leptin, inflammatory markers, and antioxidant status

Variables	Female	Male
Triglyceride (mg/dL)		
Pre	111 ± 18	117 ± 8
Post	119 ± 21	131 ± 23
Cholesterol (mg/dL)		
Pre	210 ± 11	213 ± 12
Post	209 ± 11	228 ± 12
HDL (mg/dL)		
Pre	60 ± 7	48 ± 8
Post	58 ± 7	48 ± 8
VLDL (mg/dL)		
Pre	27 ± 5	31 ± 5
Post	25 ± 5	38 ± 5
LDL (mg/dL)		
Pre	123 ± 10	132 ± 11
Post	125 ± 11	141 ± 11
Apo A (mg/dL)		
Pre	169 ± 12	154 ± 12
Post	154 ± 13	151 ± 13
Apo B (mg/dL)		
Pre	93 ± 6^b^	111 ± 7^a^
Post	91 ± 6^b^	115 ± 7^a^
Leptin (ng/mL)		
Pre	35 ± 3^a^	8 ± 3^b^
Post	32 ± 3^a^	10 ± 4^b^
Interleukin-1α (pg/mL)		
Pre	1.30 ± 0.18^c^	1.83 ± 0.09^ab^
Post	1.43 ± 0.18^bc^	2.00 ± 0.09^a^
C-reactive protein (mg/dL)		
Pre	4.16 ± 0.56	3.34 ± 0.67
Post	3.13 ± 0.56	2.35 ± 0.67
Antioxidant capacity (mM)		
Pre	0.79 ± 0.05^b^	0.83 ± 0.05^b^
Post	0.96 ± 0.06^a^	1.10 ± 0.06^a^
Malondialdehyde (μM)		
Pre	29 ± 7^b^	56 ± 8^a^
Post	13 ± 7^c^	22 ± 8^bc^

^a,c^Means with unlike superscript are significantly (P ≤ 0.05) different from each other.

## Discussion

In the present study, the lack of effects of carrot juice on cardiovascular markers may well be attributed to the fact that no change in eating pattern was requested [38]. The subjects were told to continue with their daily lifestyle and drink one glass containing 16 fl oz (480 mL) of carrot juice as a morning snack.

Despite no change in anthropometric or lipid panels, carrot juice contributed to a 5% reduction in systolic blood pressure. The trend in systolic blood pressure lowering effect of carrot juice is consistent with the National Heart, Lung, and Blood Institute's recommendations to increase intake of vegetables as part of a dietary pattern [39]. It is also consistent with the finding that systolic blood pressure, rather than diastolic, is more sensitive to nutrient intake modifications [40]. The nutrients present in carrot juice, including fiber, potassium, nitrates, and vitamin C could have contributed to the effect seen in lowering systolic blood pressure [41]. Elevated blood pressure is a primary risk factor for the development of CVD and is described as a component of the metabolic syndrome [42]. Carrots are a rich source of nitrates, which may be converted into nitric oxide to increase vasodilation, possibly decreasing blood pressure. The Mediterranean diet, known for relatively low rates of CVD compared to the typical Western diet, is rich in dietary nitrates and green leafy vegetables containing potassium [43]. Carrot juice is also a rich source of potassium which may in part have contributed to lowering systolic blood pressure [44].

The lack of effect from drinking carrot juice on the plasma inflammatory markers as well as insulin and leptin may have been because insulin, leptin, interleukin-1α, and C-reactive protein were all within normal ranges. Our report is inconsistent with an earlier finding suggesting that elevated C-reactive protein was independently associated with higher BMI and higher triglycerides [45]. However, our results are consistent with those who reported that the mean values of cholesterol, triglycerides, and LDL cholesterol were not affected by increased C-reactive protein and insulin in obese women when compared to normal weight controls suggesting that the increase in inflammatory markers have no effect on lipid panel status [46]. Therefore, we conclude that the high mean cholesterol and triglyceride concentrations are attributed to the diet and the eating habits rather than to inflammatory stimulation.

It is interesting to note that the increase in the plasma antioxidant status and the decrease in the plasma malondialdehyde may have been related to drinking carrot juice which is a good source of β-carotenes and α-carotenes with antioxidant activity. In a similar study, drinking kale juice significantly improved antioxidant status without affecting the concentration of malondialdehyde [47]. The inconsistency in results is probably due to the amount of juice consumed. In the current study, the participants consumed 16 fl oz (480 mL) of fresh carrot juice everyday while in the study reported by Kim and colleagues, the participants drank 5 ounces (150 mL) of Kale juice daily [47].

Our results support the notion that physiological differences exist between the male and female subjects. One of the physiological differences between the participants are sex hormones causing men to weigh more and have a lower body fat percentage then the female counterpart [48,49]. Furthermore, the higher plasma leptin and insulin concentrations in females agrees with earlier reports and the differences could also be attributed to sex hormones as observed in one other study [50]. However, the elevated plasma interleukin-1α, Apo B, and malondialdehyde in men suggest that men are at a greater risk for developing CVD than women. Moreover, decreased lipid peroxidation evident from drinking carrot juice is associated with increased antioxidant status independent of inflammatory markers, hormones, or increased cholesterol and triglyceride concentrations.

## Conclusion

In conclusion, drinking 16 fl oz of fresh carrot juice daily significantly increased antioxidant status and suppressed lipid peroxidation without affecting the plasma cholesterol and triglyceride status. It appears both dietary and lifestyle change are required to positively alter lipid profiles in place of increasing fruits or vegetable serving sizes without comprehensive dietary modifications. However, future studies are needed to either refute or validate the results and conclusion of this study.

## Abbreviations

TAMUK-IRB: Texas A&M University-Kingsville Internal Review Board; fl oz: Fluid ounce; BMI: Body mass index; CVD: Cardiovascular disease; LDL: low-density lipoprotein; HDL: High density lipoprotein; Apo A: Apolipoprotein A; Apo B: Apolipoprotein B; NHLBI: National Heart, Lung, and Blood Institute; TLC: Therapeutic lifestyle change; LRC-CPPT: Lipid Research Clinics Coronary Primary Prevention Trial; BIA: The Bioelectrical impedance analysis; DNA: Deoxyribonucleic acid; SAS: Statistical analysis software.

## Competing interests

The authors declare that they have no competing interests.

## Authors' contributions

ASP coordinated and implemented the study, conducted laboratory analysis, and helped to draft the manuscript. SF conducted laboratory analysis. AS drafted the manuscript. BSP designed the study and drafted the manuscript. FD designed and coordinated the study, performed the statistical analyses, and helped to draft the manuscript. All authors read and approved the final manuscript.
